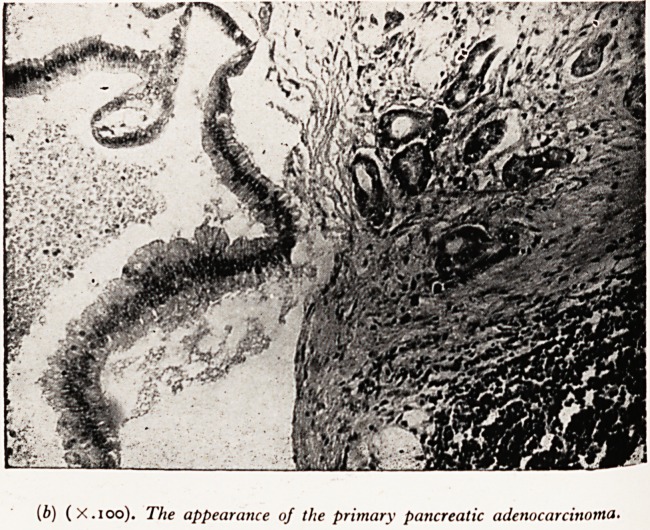# Alveolar Cell Carcinoma of Lung

**Published:** 1959-07

**Authors:** T. F. Hewer


### ALVEOLAR CELL CARCINOMA OF LUNG: A CASE ATTRIBUTED TO METASTASIS FROM A PANCREATIC CARCINOMA

*A Clinical Pathological Conference of the University of Bristol Medical School.*

CHAIRMAN: PROFESSOR T. F. HEWER

*Professor T. F. Hewer:* I will ask Dr. Pearson who looked after this patient to give us the clinical history.

*Dr. J. E. G. Pearson:* This lady was 66 when I first saw her in 1955. She was referred to me as "post-operative chest", nine months after she had had an arthroplasty operation for osteoarthritis of the left hip. No chest X-ray had been taken at that time so there was no chance of assessing whether or not it really was a "post-operative chest". Her main symptoms were cough and shortness of breath, which she originally dated from her operation, but a student, on fuller questioning, ascertained that she had, in fact, been breathless for a year before that.

She came into hospital with a cough, a fair amount of mucoid sputum—between 2 to 3 ounces each day—and her dyspnoea; she had a little chest pain of paradyspnoeic type. There had been no haemoptysis.

She was a healthy looking woman for her age. She became breathless rather easily after walking about in the ward.

On her first admission she had very few signs indeed in her chest but did have increased, almost bronchial, breathing in patchy distribution on both sides. This corresponded with the X-ray appearance of several consolidated areas. She was put on physiotherapy. She had not brought up any pus to speak of, and the pathologists found no specific organisms in the sputum: she improved a little and went out. I followed her up in Outpatients and throughout this time the X-ray appearance was slowly progressive and her symptoms certainly got slightly worse.

I took her in again the following year (1957) for another check-up, and to see if we could do anything more, hoping to make her well enough for bronchogram or bronchoscopy, but she remained too breathless to contemplate either investigation. She looked at me pathetically, as though we were doing little to help her—and in fact we weren't doing much! Throughout this time there were no abnormal cardio-vascular signs. Even as late as six months before death an E.C.G. was perfectly normal. With her lung picture we were always waiting for the onset of cor pulmonale and I would emphasize that there were no signs of bronchial obstruction. Later she developed numerous rhonchi, broncho-spasm and a few rales at the bases.

An incidental note is that she had haematuria. She had already been under Mr. Pocock because of papilloma of the bladder in 1944. This recurred and was cauterized. Intravenous pyelogram was carried out and the radiologist reported a possible filling defect in the right kidney, so that we considered the possibility of kidney neoplasm with pulmonary metastasis.

On her final admission two weeks before death she was very breathless and given oxygen. She showed no swelling of the ankles or signs of infection. Acute cor pulmonale was probably the immediate cause of her death.

*Dr. J. H. Middlemiss* (Demonstrating the radiographs). The first X-ray shows advanced osteoarthritis of her left hip. The second shows arthrodesis by central dislocation. I.V.P. reveals that both kidneys were functioning well. The possibility of a renal lesion was raised but in fact the apperance of these kidneys is within normal limits. The bladder appears normal and there is no evidence of bladder tumour, but then they have to be very big indeed to show radiologically.

The third X-ray shows her chest, the condition with which she came to Dr. Pearson. In the right upper lobe is an area of consolidation, solid lung not collapsed but de-aerated. All alveolar spaces are filled with something. There is a further similar area in the right lower lobe, with the middle lobe aerated. On the left side the lingula and probably left lower lobe are affected.

The fourth X-ray shows her chest condition a year later. There is still solid lung in the right upper lobe with aeration at the apex of the lower lobe—not a great change. We discussed the possibilities and put forward such ideas as chronic inflammatory change and progressive massive fibrosis. The radiologists did not think this appeared to be neoplastic in view of the long interval of time and the lady's static condition.

The fifth X-ray was taken a year later: the process is still there. Upper lobe still solid; contraction of lower lobe with an irregular edge. Solid lung on the left. Compensatory emphysema of the middle lobe on the right. This was within 3 months of death.

Altogether there was not a great deal of change over a long period, the main feature being contraction of the process, getting smaller rather than bigger, so neoplasm was not a diagnosis one would put forward with any conviction. My view was that this process was fibrotic.

*Professor Hewer:* At autopsy she looked quite well nourished. There was the scar of the successful arthroplasty operation over the left hip. The neck veins were dilated and there was cyanosis: no clubbing of the fingers. There was no tumour in the breasts. The left pleural cavity contained a little and the right 200 ml. of clear fluid with no adhesions.

The trachea contained some extraordinary tenacious white mucus but there was no sign of hyperaemia; this absence of any indication of inflammation of the trachea was surprising because the lungs seemed to me, on external examination, to be full of pneumonic exudate with areas of organisation: that was my impression before I sectioned the lungs. On the other hand it did seem odd that there were no pleural adhesions. A culture from the trachea yielded no pathogenic organisms.

The lungs themselves presented a remarkable appearance and were heavy and almost completely airless. Large parts of all lobes were evidently full of gelatinous mucus but some firm nodular areas were also palpable and there were some deep scarred clefts on the pleural surface suggesting the presence of old areas of collapse. No tumour was visible externally and the hilar and mediastinal lymph nodes showed no gross sign of tumour or of enlargement. On section after fixation by injection through the trachea (Plate XXXII(*a*)) there were large areas of gelatinous consolidation, having in places a clear-cut lobular outline, and within these areas were smaller irregular firm grey masses that were clearly neoplastic and probably, judging by their opacity, carcinomatous.

In places there were old emphysematous bullae and it looked as though the neoplasm had invaded a lung that was already emphysematous. Nowhere could any tumour be found in the walls of any of the bronchi and there was no particular area of tumour that could be considered the starting point of the process. Many of the bronchi were completely filled with the tenacious clear mucus and many lobules were consequently collapsed but they were mostly filled with mucus and their alveolar outlines were still apparent. Large areas of the lung were quite translucent although solid.

From the gross appearance of the cut surface it was obvious that the firm grey areas were tumour and the tentative diagnosis of organized pneumonia was abandoned. The lymph nodes were not enlarged and contained no gross tumour metastases.

As there was a history of recurrent bladder papilloma a careful examination was made of this lesion; the bladder still contained a few small granular masses of tumour in the mucosa above the trigone but, to anticipate the results of microscopy, these were a transitional cell carcinoma bearing no resemblance to any of the cells in the lung.

The only other site of tumour was in the pancreas where, at the inferior margin of the centre of the body, was a hard scirrhous mass 3 cm. in diameter with some cystic areas containing tenacious mucus.

I came to the provisional diagnosis of alveolar cell carcinoma of the lung with a metastasis in the pancreas. It was clear that the bladder carcinoma had nothing to do with it.

The only significant changes in other organs were some hypertrophy of the right ventricle of the heart and dilatation and atheroma of the pulmonary artery, together indicating the presence of pulmonary hypertension. It is noteworthy that the kidneys were normal.

Microscopically, the solid grey areas in the lungs were due to a well differentiated adenocarcinoma of high columnar cells lining the alveolar spaces and often filling the lumen entirely, without damaging the walls (Plate XXXIII(*b*)). These columnar tumour cells were mucin-producing and accounted for the tremendous quantity of firm tenacious mucus that filled all the adjacent alveoli and quite a number of lobules. In the mucus were many macrophages (Plate XXXII(*a*)). There was no suggestion of scarring of the alveolar walls; everywhere they were intact and had the full content of elastic fibres. Some of the respiratory ducts were lined by normal respiratory mucosa but many had an adenocarcinomatous lining and so had some of the emphysematous bullae.

A single small metastasis was found microscopically in one of the hilar lymph nodes. The liver, spleen, kidneys, adrenals and mediastinal and abdominal lymph nodes, the vertebrae and ribs that were examined showed no tumour.

The most interesting feature of the histological study was provided by the tumour in the pancreas. This proved to be a primary adenocarcinoma of pancreas with some well differentiated areas closely resembling the pancreatic ducts (Plate XXXIII(*b*)). In places the tumour had entered lymphatic vessels and was also invading some small veins. It was indistinguishable from the tumour in the lungs and it became apparent that this was a case of primary carcinoma of the body of the pancreas with extensive pulmonary metastases invading the alveolar spaces and utilizing the alveolar walls as supporting structures.

Having disposed of the diagnosis of primary alveolar carcinoma of lung in this case I looked through our post-mortem records of the last twenty years and found that we had only two previous cases indexed as examples of this condition. They seemed worth examining critically.

The first of these was a man of 65 and a piece of his lung was mounted in our museum as an example of alveolar carcinoma of the lung. There was a massive carcinoma involving a large part of one lung. The main bronchi within this lung were reported as being completely destroyed and unrecognizable. Microscopically it was a pleomorphic carcinoma, evidently bronchogenic in origin. Metastases were present in both suprarenals, liver and para-aortic lymph nodes. There was no real justification for calling this an alveolar carcinoma: it was a bronchogenic carcinoma invading the lung, filling the alveolar spaces.

The second case was a man of 39 whose lungs had diffuse carcinomatous infiltration with groups of intact alveoli lined by well differentiated columnar carcinoma cells. In this case there was also a small scirrhous carcinoma in the head of the pancreas and at the time there was great debate as to whether the primary was in the lung or in the pancreas. The metastases were much more widespread than in our present case. On reviewing the histological material there is now little doubt that it also was an example of primary carcinoma of pancreatic ducts with pulmonary metastases.

There have been several reviews recently of alveolar cell carcinoma of lung, which is sometimes called pulmonary adenomatosis, (Storey et al. 1953, Farber et al. 1954) most of which accept fully the idea that it is a multicentric tumour derived from the alveolar epithelium, or that of the respiratory ducts. Johansen and Olsen (1957), however, in their review of the subject report twelve cases of their own, one of which was due to a primary tumour of the pancreas; they mention that Eck (1955) doubted the existence of primary alveolar carcinoma of the lung. He actually reported three cases in which there was intra-alveolar spread of carcinoma of columnar cell type; one was

PLATE XXXII

(*a*) *Section through the lungs, showing areas of gelatinous consolidation and areas of growth.*

(*b*) (×.100). *Section of lungs, showing a well differentiated columnar cell adenocarcinoma lining the alveolar spaces.*

(*a*) (×.100). *Showing an area of the lung tumour in which there is copious mucin formation.*

(*b*) (×.100). *The appearance of the primary pancreatic adenocarcinoma.*

a metastasis from a primary pancreatic carcinoma, one from the rectum and one from a bronchus invading the lung.

*Dr. Pearson:* In seeking for a rare diagnosis for this unusual case I had thought of so-called alveolar cell carcinoma of the lung as quite a possibility: there are two main patterns of carcinoma of this type described; one is that of multiple small scattered areas sometimes mistaken microscopically at autopsy for bronchopneumonia, while the other shows one massive area. I reckoned that this case might be a mixture of the two forms.

The origin of alveolar cell carcinoma has always been in some doubt and the most popular view now is that it is a type of bronchiolar growth.

*Dr. J. E. Cates:* You said, Professor Hewer, that the tumour was only 3 cms. across, and yet it antedated the change in the lungs which had been present for two years. Is this all right from a pathological point of view?

*Professor Hewer:* Yes, it must have remained small for a long time—an extraordinarily long time—but well differentiated tumours like this may be very slow: there was a dense fibrous reaction around it.

*Dr. Cates:* Does the average carcinoma of the pancreas secrete as much mucus as that, or was it a peculiarity of metastases in the lungs?

*Professor Hewer:* No, they do not usually do so, but any tumour from a mucin-secreting mucosa may secrete a great deal of mucus.

*Dr. A. B. Raper:* I believe this tumour arose from the pancreatic duct.

*Professor Hewer:* Yes, it did and I think that may well be the explanation of the excessive mucin production. In conclusion I should like to stress the point that we ought to be very critical of any case of so-called alveolar cell carcinoma of lung and make an exhaustive search for an alternative primary.

### REFERENCES

Eck, H. (1955), *Zeitschr. f. Krebsforsch.*, **60**, 433.

Farber, S. M., Wood, D. A., Sangalli, F., Pharr, S. L. and Rukmono, M. D. (1954). *Surg. Gyn. and Obst*, **99**, 483.

Johansen, C. and Olsen, S. (1957). *Acta Path. et Microbiol. Scand*, **41**, 187.

Storey, C. F., Knudtson, K. P. and Lawrence, B. J. (1953). *J. Thorac. Surg.*, **26**, 331.

## Figures and Tables

**(a) f1:**
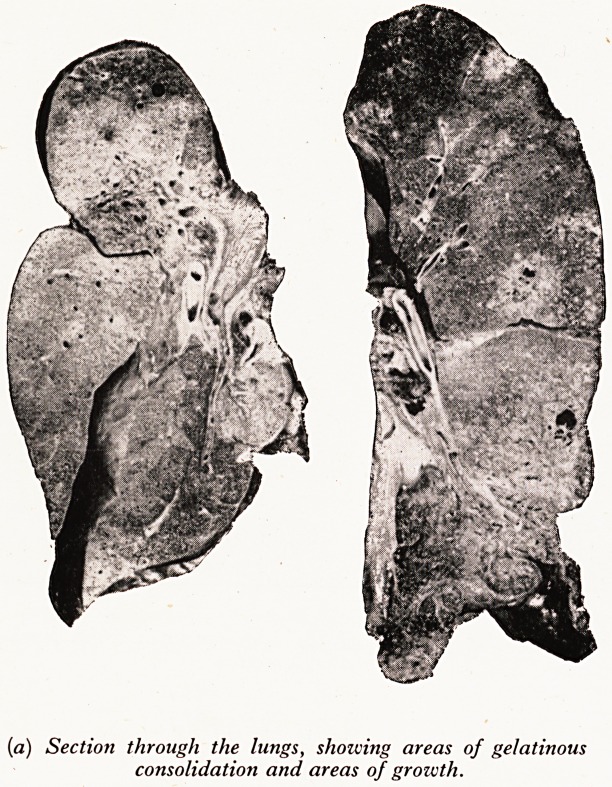


**(b) f2:**
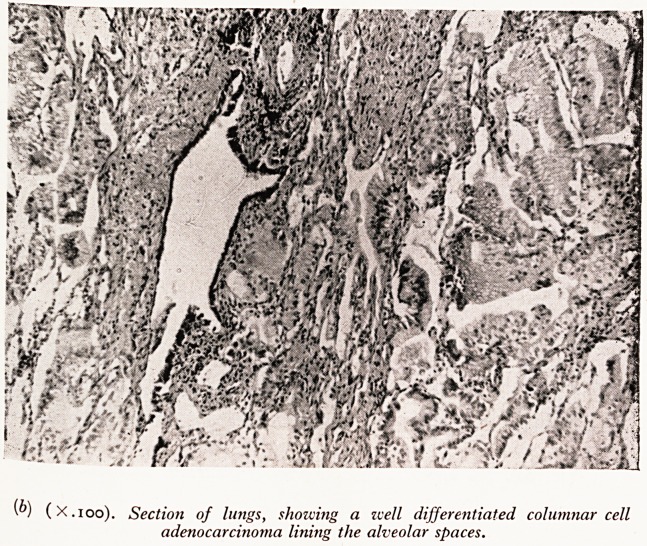


**(a) f3:**
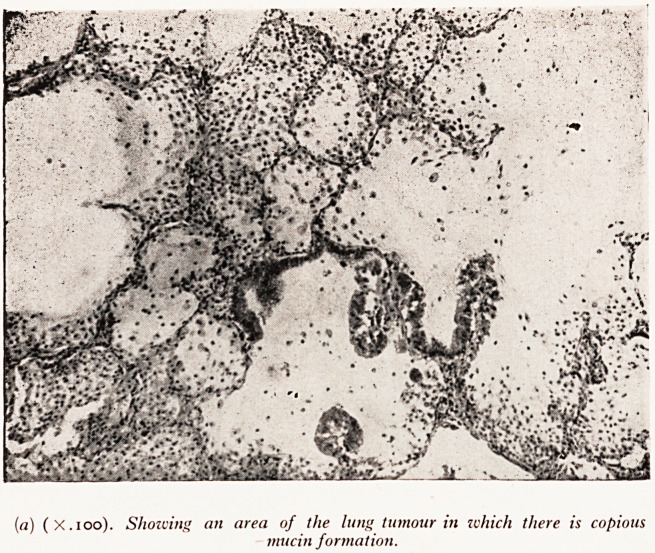


**(b) f4:**